# Charting the role of the number line in mathematical development

**DOI:** 10.3389/fpsyg.2013.00641

**Published:** 2013-09-18

**Authors:** Jo-Anne LeFevre, Carolina Jimenez Lira, Carla Sowinski, Ozlem Cankaya, Deepthi Kamawar, Sheri-Lynn Skwarchuk

**Affiliations:** ^1^Institute of Cognitive Science, Carleton UniversityOttawa, ON, Canada; ^2^Department of Psychology, Carleton UniversityOttawa, ON, Canada; ^3^Faculty of Education, University of WinnipegWinnipeg, MB, Canada

**Keywords:** number line, spatial abilities, number system knowledge, arithmetic, mathematics

## Abstract

Individuals who do well in mathematics and science also often have good spatial skills. However, the predictive direction of links between spatial abilities and mathematical learning has not been firmly established, especially for young children. In the present research, we addressed this issue using a sample from a longitudinal data set that spanned 4 years and which includes measures of mathematical performance and various cognitive skills, including spatial ability. Children were tested once in each of 4 years (Time 1, 2, 3, and 4). At Time 3 and 4, 101 children (in Grades 2, 3, or 4 at Time 3) completed mathematical measures including (a) a number line task (0–1000), (b) arithmetic, and (c) number system knowledge. Measures of spatial ability were collected at Time 1, 2, or 3. As expected, spatial ability was correlated with all of the mathematical measures at Time 3 and 4, and predicted growth in number line performance from Time 3 to Time 4. However, spatial ability did not predict growth in either arithmetic or in number system knowledge. Path analyses were used to test whether number line performance at Time 3 was predictive of arithmetic and number system knowledge at Time 4 or whether the reverse patterns were dominant. Contrary to the prediction that the number line is an important causal construct that facilitates learning arithmetic, no evidence was found that number line performance predicted growth in calculation more than calculation predicted number line growth. However, number system knowledge at Time 3 was predictive of number line performance at Time 4, independently of spatial ability. These results provide useful information about which aspects of growth in mathematical performance are (and are not) related to spatial ability and clarify the relations between number line performance and measures of arithmetic and number system knowledge.

## Introduction

Individuals who do well in mathematics and science also usually have good spatial skills (Wai et al., 2009). However, causal links between spatial ability and mathematical learning have not been firmly established, especially for young children (Mix and Cheng, 2012). Thus, despite substantial correlational evidence for links between these two cognitive domains, especially for older children and adults (Mix and Cheng, 2012), a large amount of research remains to be done explaining the nature and direction of the links. In recent research, it has been suggested that a specific skill, that is, estimating the location of numbers on a number line, mediates the link between spatial abilities and growth in conventional mathematical knowledge (Gunderson et al., 2012). The goal of the present research was to use longitudinal data to test the predictive pathways among spatial abilities, number line task performance, and mathematical learning for children in elementary school.

It seems relatively uncontroversial that spatial tasks, especially those tapping visual-spatial working memory or mental rotation, are correlated with mathematical task performance (Mix and Cheng, 2012). One type of mathematical task, the number line task, has received a great deal of attention in this regard. For the number line task, children are shown a line with the left end marked as 0 and the right end marked with a number such as 10, 100, or 1000 (Siegler and Opfer, 2003; Siegler and Booth, 2004; Booth and Siegler, 2006; Laski and Siegler, 2007). In the number-to-position version of the task, children are shown or told a number (e.g., 47) and asked to indicate its location on the number line (Laski and Siegler, 2007). In the position-to-number version, they are asked to estimate the number indicated by a marked position on a line (Siegler and Opfer, 2003; Ashcraft and Moore, 2012). Children above 6 years of age appear to understand the requirements of the task and it is relatively simple and easy to administer. In many studies over the last 10 years, performance on the number line task has been shown to correlate with various standardized mathematical performance measures and with assessments of measurement, numerosity, and computational estimation (Siegler and Booth, 2004; Booth and Siegler, 2006).

**Figure 1:**
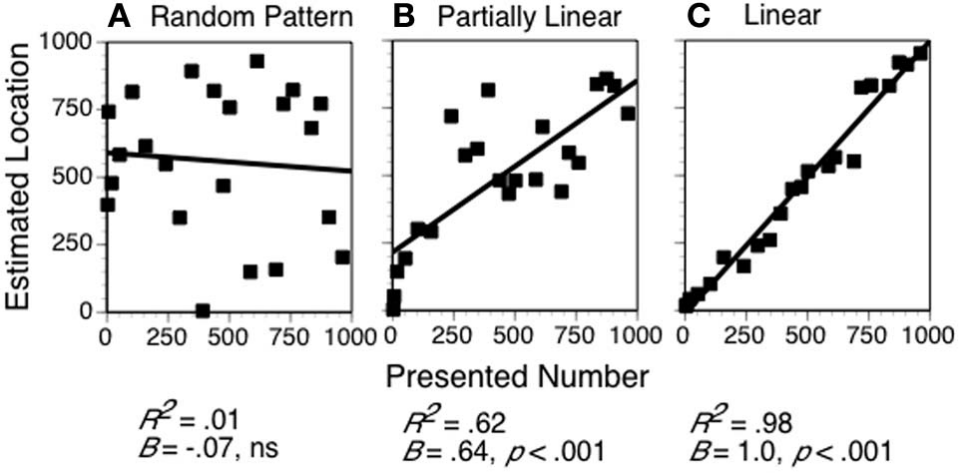
**Examples of different forms of the relation between numbers presented and locations selected in the number line task, (A) random pattern of responses, (B) partially linear pattern, and (C) linear pattern**. Data are from three participants from grade 2 in the current study. The model fits and slope values are shown below the graph.

The number line task seems ideal for examining the links between spatial abilities and mathematical learning because it requires knowledge and processes from both domains. Children presumably must be relatively familiar with the number system in the range specified in the particular version of the number line task, and they need to use proportional reasoning skills (or some other strategy) to connect the number to the position on the line (or vice-versa). Ashcraft and Moore (2012), using a position-to-number version of a 0–1000 number line, showed that children from Grades 3 to 5 and adults used an implicit midpoint reference to bisect the line and guide their number choices, and thus, showed very good performance for locations close to 500. The adults and older children also showed good performance (i.e., less variability) for locations close to 250 and 750, suggesting they were using a proportional reasoning strategy of dividing the line into quarters. These data support the view that adults and older children use proportional reasoning strategies to make decisions about locations on the number line (see Barth and Paladino, 2011). For younger children, data collected by Moeller and colleagues (2009) with Grade 1 children and by Bouwmeester and Verkoeijen (2012) with children in kindergarten, grade 1, and grade 2 suggested that many children use a counting-based strategy that results in relatively ordinal and linear performance at the smaller end of the number line, with variable performance for the end, resulting in either logarithmic fits or patterns described by two separate lines. Examples of some patterns of performance are shown in Figure 1 for children in Grade 2 from the current sample.

A few children do poorly on this task, as shown by the pattern in Figure 1A. In contrast, most children at this age show some understanding of the relative position of the numbers on the line. For example, the child whose data are shown in Figure 1B produced ordinally-correct positions that are overestimates below 250, and relatively uniform (and thus, non-ordinal) responses for the larger numbers. Depending on the exact shape, this pattern is fit better with logarithmic, quadratic, or exponential functions; however, the linear fit in this case is also statistically significant. Moeller et al. (2009) showed that two different regression lines, one for the numbers in the lower range and one for the numbers in the higher range, also provide a good fit for many children showing this pattern (see Bouwmeester and Verkoeijen, 2012). One interpretation of this pattern is that these children have relatively intact number knowledge up to a certain point, but a weak grasp of the larger numbers and their inter-relations. Finally, consider the highly accurate linear pattern shown in Figure 1C. Individuals whose performance shows a linear pattern presumably have a strong grasp of the ordinal positions of numbers, including the uniform spacing of the numbers in the indicated range, and sufficient spatial skills to produce very accurate positioning. One goal of the present study was to examine how children's estimates changed over time in relation to their spatial and numerical skills.

Some researchers (Siegler and Opfer, 2003; Siegler and Booth, 2004; Booth and Siegler, 2006; Laski and Siegler, 2007) have interpreted less linear patterns as representative of a non-linear internal mental representation for magnitude rather than a reflection of different strategic processes (cf. Moeller et al., 2009; Bouwmeester and Verkoeijen, 2012). The view of number line performance as an index of children's internal representation of magnitude has not been conclusively proven and is not a necessary assumption: Number line task performance is an interesting and relevant measure even if it does not reflect an internal mental number line. Strong claims about number line performance as a reflection of an internal representation have been based on finding high correlations between number line performance and other mathematical tasks (Booth and Siegler, 2008) or on the increasing linearity of the patterns that children produce as they get older (Booth and Siegler, 2006). More recently, research showing that the strategies children adopt on the task are strongly related to their patterns of performance (Moeller et al., 2009; Bouwmeester and Verkoeijen, 2012) suggest that it is not necessary to postulate direct connections to an internal mental number line to understand performance. Regardless of the interpretation of number line task performance that is assumed, the data show that younger children tend to produce less linear number line estimations than older children. Thus, progress toward linear performance on the number line task can be used as an index of growth in children's understanding of the symbolic number system. Because placement of targets on the number line is a necessary component of the task, we hypothesized that spatial reasoning abilities would be related to growth in number line performance. Importantly, however, we also expected that children's knowledge of the number system would influence their developing number line performance.

We know of only one other study that evaluated changes in number line task performance over time in relation to both spatial skill and mathematically-relevant knowledge. Gunderson et al. (2012; Experiment 1) had children complete a measure of spatial processing (mental rotation) at the beginning of first or second grade (*N* = 152). They also completed a 0–1000 number line task at both time points. At the end of the year, they completed a measure of arithmetic problem solving. Gunderson et al. found that spatial ability predicted growth in number line performance across the year, as did the arithmetic measure. These results were the first to show that spatial ability is linked to improvements in children's number line task performance. In a second study, Gunderson et al. investigated whether number line task performance would predict later mathematical achievement. In this study, 42 children completed spatial measures at age five, a 0–100 number line task at age six, and an approximate symbolic arithmetic task at age eight. Performance on all three measures were correlated, however, the relation between spatial ability and approximate arithmetic was completely mediated by number line task performance. Gunderson et al. suggested that their findings supported causal links between early spatial ability, acquisition of a linear number line, and later number knowledge. Another goal of the present research was to further test this proposed causal chain and to examine whether this finding holds for older children than previously tested.

In the present research, longitudinal data from a large study of children's early mathematics development was used to evaluate three hypotheses. First, we hypothesized that spatial ability would be correlated with number line performance, as well as with traditional measures of mathematical performance (i.e., arithmetic and number system knowledge). Second, we hypothesized that spatial ability would predict growth (change over time) in number line task performance, above and beyond its relation with other mathematical skills. This hypothesis was based both on the findings of Gunderson et al. (2012) and on the assumption that the number line task requires explicit spatial processing in the form of proportional reasoning (Ashcraft and Moore, 2012). Third, we tested the predictive pathways between number line task performance and several other mathematical measures. Using cross-lag panel analysis, we assessed the longitudinal relations between number line task performance and measures of mathematical performance. These analyses provide a stringent test of the hypothesized directional links between number line task performance and (for example) arithmetic, because performance measures were available longitudinally for all measures.

## Methods and materials

### Participants

The participants were part of a longitudinal study of over 500 children that spanned 4 years (see LeFevre et al., 2010 for analyses of a younger cohort from the same study; also Kamawar et al., 2010; LeFevre et al., 2013). Children were tested once each year (i.e., Time 1, 2, 3, and 4). Data from 101 children (55 girls; 46 boys) who had completed the number line task twice (at Times 3 and 4 of the project) were analyzed in this paper. At Time 3 the participants were in Grade 2, 3, or 4 (*n*s of 52, 27, and 21). The mean ages were 7:10, 8:10, and 9:10 (in years: months) at Time 3 for children in grades 2, 3, and 4, respectively. Children were recruited from several different schools in two Canadian cities. Eighty-two of the children were monolingual English speakers and the others spoke another language in addition to English. The majority of children came from two parent families and most parents had education beyond high school. Thus, the sample was predominantly middle class.

### Measures

Children completed a range of cognitive and mathematical measures. More detail about each measure is provided below. In the present analysis, we used measures of (a) spatial ability, (b) number line estimation, (c) number system knowledge, and (d) arithmetic. Control variables included vocabulary, grade, and gender. The mathematical measures were completed by the children twice (Years 3 and 4 of the study). The mathematics measures utilized in this study were chosen to represent typical symbolic number and arithmetic tasks.

#### Spatial ability

Children were administered the Analogy subtest of the Cognitive Intelligence Test Nonverbal (CIT; Gardner, 2000) either at Time 2 (children from Grade 2 or 4 group) or Time 3 (children from Grade 3 group) of the study. On each trial, children were shown six squares with visual patterns in only five of the squares. They are then asked to pick the “missing pattern” from a selection of patterns at the bottom of the page. This task requires the use of analogical reasoning, mental rotation, and spatial processing for the child to identify the form or design that best completes the pattern. Standard scores on this test have a mean of 100 and a standard deviation of 15. Scaled scores have a mean of 10 and a standard deviation of 3. The test manual reports that the reliability for this subtest was calculated using the Kuder-Richardson formula at each one year age level: for 6, 7, and 8 year-olds reliability was 0.82; for the 9 year-olds the reliability was 0.75. Three children were missing a score so these were replaced with the overall mean for the task.

Children also completed a spatial memory span measure that was administered on a laptop computer. Similar spatial span measures have been used in other studies (e.g., Berch et al., 1998; Rasmussen and Bisanz, 2005). In this task, after the children pressed the “GO” button, a set of nine green circles (lily pads) appeared on the screen and the children watched a cartoon frog as it jumped from one lily pad to another at 1 s intervals. After viewing a sequence of “jumps”, the child was given a pointer and asked to reproduce the sequence. As the child pointed to each location on the screen, the experimenter moved the cursor to the corresponding location and clicked on it so the sequence was saved in the computer.

Children completed one practice trial, during which the experimenter watched the frog and then pointed to the two locations in sequence. The test trials consisted of two sequences for each length with the spans increasing in length by one after each pair. The test trials began with two locations and went up to eight. The task ended when the child made errors on two consecutive trials at a specific length. For the analysis, data was used for this task from Time 2; however, Time 1 data were used for 10 children who were missing these data at Time 2. The score was the total number of sequences correct (maximum score of 14). The scores were standardized by creating *z*-scores by age group. The split-half reliability of this task was 0.78 based on the sum of the first and second trial at each length.

#### Numeration

Numeration was assessed using the numeration subtest of the Key Math Test-Revised (Connolly, 2000). This Canadian norm-referenced test has two alternate forms; for this study Form B was administered at Time 3 and Form A was administered at Time 4. Children typically attempted between 18 and 30 items on this task.

This task assesses children's knowledge of the number system by having them name numbers and demonstrate understanding of the ordering of symbolic quantities and an understanding of place value for numbers between 100 and 1000. For example, they may be asked to put three numbers in order. Raw scores were used for the analyses; standardized scores on this test have a mean of 10 and a standard deviation of 3. The alternate form reliability coefficient for the numeration subtest within an American sample was 0.75 (Connolly, 2000).

#### Calculation

Children completed the Calculation subtest of the Woodcock-Johnson Tests of Achievement—Revised (Woodcock and Johnson, 1989). During this paper and pencil test, the children solved mathematical problems such as 3 + 4 or 15 − 8 presented in order of increasing difficulty. Testing was stopped after the child incorrectly answered six questions in a row. The score is standardized by grade. Split half reliabilities were reported as 0.93 and 0.89 for children aged 6 and 9, respectively. Children typically attempted between 20 and 32 items on this task.

#### Number line task

This study implemented a computerized version of the number line task introduced by Siegler and Opfer (2003). The task was described as a game called “Number Line Road.” The child was shown a straight line with 0 on its left end and 1000 on its far right end. After the child pressed the “GO” button, a number appeared on the upper right of the screen and the child had to use the mouse to place the cursor—which appeared as a red car with a red straight line beneath it—at the spot where the child estimated the number to be located along the road. When the child clicked on a location the car's last location was retained briefly and then a car horn sounded to indicate a successful placement.

Children were given three practice trials on which they had to place the car on “stop lines” located at 500, 0, and 1000 on the number line road. The test stimuli at Time 3 were those used by Booth and Siegler (2006), and included 22 trials using the following numbers: 3, 7, 19, 52, 103, 158, 240, 297, 346, 391, 438, 475, 502, 586, 613, 690, 721, 760, 835, 874, 907, 962. The order of presentation of the stimuli was randomized separately for each child. The stimuli used at Time 4 were those used by Laski and Siegler (2007). They included 25 trials that were selected to evenly distribute the numbers across the number line. Thus, they included four numbers between 0 and 100, four numbers between 900 and 1000, two numbers from each other decade and distances matched from the endpoints. The numbers used were: 6, 18, 59, 97, 124, 165, 211, 239, 344, 383, 420, 458, 500, 542, 580, 617, 656, 761, 789, 835, 876, 903, 991, 982, 994. A linear regression was run for every child to determine the *R*^2^ and linear slope of the fit between actual and estimated locations. Reliability coefficients for the initial larger sample of children computed based on split-half of odd and even trials at Time 3 and Time 4 were 0.856 and 0.866 (Cronbach's alpha, ns of 203 and 238).

#### Receptive vocabulary

At Time 1, all children completed the Peabody Picture Vocabulary Test—Third Edition (PPVT III; Dunn and Dunn, 1997) as a measure of their receptive vocabulary. During this test, children were shown a set of four pictures and asked to select the picture that best suits the target word presented by the examiner. This test is norm-referenced and is standardized by age; it has a mean of 100 with a standard deviation of 15. Reliability for this test is reported as 0.94 (Cronbach's alpha).

### Procedure

Children were tested during two one-on-one 30-min sessions, or in one 60-min session, that took place within the children's schools. The standardized tests were completed in one session and the computer adapted tests were administered during the other session. Each session was conducted by a different experimenter. The computer adapted tests were administered on a laptop computer. The keyboard was covered except for the spacebar which acted as the “GO” button. In order to have the child's attention on the screen when the stimuli appeared, the child was asked to initiate each trial by pressing the “GO” button.

## Results

### Descriptive statistics

Means, standard deviations, and skew for the measures are shown in Table 1. For ease of comparison, the standard scores are shown in the table for the Calculation and Numeration measures; however, raw scores for these variables had a larger range than the standard scores and thus, were used in the correlational and regression analyses.

**Table 1:** **Descriptive statistics for predictor and outcome measures**.

**Measures**	**Time 1, 2 or 3**^**a**^			**Time 4**		
	***M***	***SD***	**Skew**	***M***	***SD***	**Skew**
**COGNITIVE SKILLS**						
Vocabulary	109.9	10.4	−0.223			
Spatial reasoning	104.4	13.8	−0.623^**^			
Spatial span	6.0	1.9	0.312			
**MATHEMATICAL SKILLS**						
Numeration scaled	12.8	2.8	−0.240	11.0	3.3	−0.272
Numeration raw	16.5	3.4	−0.091	17.4	3.4	−0.230
Calculation standard	96.6	14.0	−0.078	91.2	14.6	0.307
Calculation raw	14.2	4.0	0.168	16.5	4.7	0.682^**^
**NUMBER LINE**						
Linear slope	0.64	0.19	−0.701^**^	0.74	0.20	−0.275
Linear *R*^2^	0.76	0.21	−1.705^***^	0.85	0.16	−1.618^***^
Arcsine linear *R*^2^	2.18	0.53	−1.551^***^	2.44	0.43	−1.129^***^

Vocabulary, spatial reasoning, and calculation are standard scores with means of 100 and standard deviations of 15. Numeration scores are standardized with a mean of 10 and a standard deviation of 3.

** p < 0.01;

*** p < 0.001.

a Cognitive skills were assessed either at Time 1 (Vocabulary), Time 2 (Spatial span for all children, Spatial reasoning for youngest and oldest groups) or Time 3 (Spatial reasoning for middle group) whereas all reported mathematical skills were assessed at Time 3 and Time 4.

In many previous studies using the number line task, the performance measure has been the *R*^2^ value for the linear fit between presented numbers and number line locations for each participant. However, as shown in Table 1, the linear *R*^2^ value is highly negatively skewed for this age group on a 0–1000 number line. Calculating the arcsine transformation of the *R*^2^ values (Gunderson et al., 2012) reduced the skew somewhat, but it was still substantial. In contrast, although the slope value for the linear fit is still significantly skewed at Time 3, with *z* = 2.92, at Time 4 the skew is reduced and no longer significant. Accordingly, the linear slope value was used in the regression and cross-lag analyses. The slope value approaches 1.0 as linearity increases. Slopes greater than 1 are possible but were infrequent (one at Time 1 of 1.08; eight at Time 2 with the largest 1.09). Thus, slopes were used to index number line performance. They capture both the absolute and relative accuracy of children's number line performance. Linear *R*^2^ could be very high as long as the ordinal positions of the numbers were preserved, but slopes will continue to improve as the locations are placed more accurately (i.e., when children are neither under nor overestimating at the ends of the range). We used slope as the index of number line performance rather than an accuracy measure (e.g., percentage of absolute error) because we wanted the dependent variable to be similar to that used by Gunderson et al. and by Siegler in most of his studies.

For spatial ability, a composite variable was calculated using principal components analysis with Spatial reasoning and Spatial Span. The factor score was used in all subsequent analyses (hereafter referred to as the Spatial Factor). It accounted for 69.8% of the variance and the two measures loaded at 0.83 on the factor.

Correlations among the measures are shown in Table 2. Correlations were based on raw scores for the mathematics measures because the range and variability was greater for raw than standard scores. Thus, grade was correlated with all of the unstandardized measures, as expected. For all measures (number line, Numeration, and Calculation), older children had better performance than younger children. Boys had higher scores on the number line measures than girls. Vocabulary was correlated with all of the other measures except for calculation. Therefore, grade, gender, and vocabulary were controlled in the analyses of number line task performance (partial correlations are shown above the diagonal). Performance was correlated at 0.70 or higher from 1 year to the next for the number line, Numeration, and Calculation measures, suggesting considerable stability of these measures. The control variables generally moderated the correlations. Because there was not enough data to evaluate patterns of performance for each grade separately, analyses were done across grades and grade was included as a control variable.

**Table 2:** **Correlations among predictors and outcomes; simple correlations below the diagonal; partial correlations above the diagonal (controlling for sex, grade, and vocabulary)**.

	**Control variables**			**Spatial factor**	**Number line**		**Numeration**		**Calculation**	
	**Grade**	**Sex**	**Voc**		**T3**	**T4**	**T3**	**T4**	**T3**	**T4**
Sex	−0.08									
Vocabulary	0.07	0.25^*^								
Spatial factor	0.12	0.01	0.24^*^		0.27^**^	0.43^**^	0.39^**^	0.32^**^	0.38^**^	0.24^*^
Number line T3	0.44^**^	0.29^**^	0.27^**^	0.31^**^		0.56^**^	0.25^*^	0.17	0.31^**^	0.28^**^
Number line T4	0.43^**^	0.36^**^	0.27^**^	0.43^**^	0.71^**^		0.48^**^	0.32^**^	0.41^**^	0.35^**^
Numeration T3	0.64^**^	0.15	0.37^**^	0.41^**^	0.53^**^	0.66^**^		0.52^**^	0.42^**^	0.36^**^
Numeration T4	0.49^**^	0.14	0.24^*^	0.36^**^	0.42^**^	0.52^**^	0.70^**^		0.38^**^	0.47^**^
Calculation T3	0.70^**^	0.00	0.13	0.36^**^	0.51^**^	0.57^**^	0.68^**^	0.59^**^		0.52^**^
Calculation T4	0.72^**^	0.13	0.14	0.26^*^	0.54^**^	0.58^**^	0.68^**^	0.66^**^	0.76^**^	

* p < 0.05;

** p < 0.01.

### Path analyses

**Figure 2:**
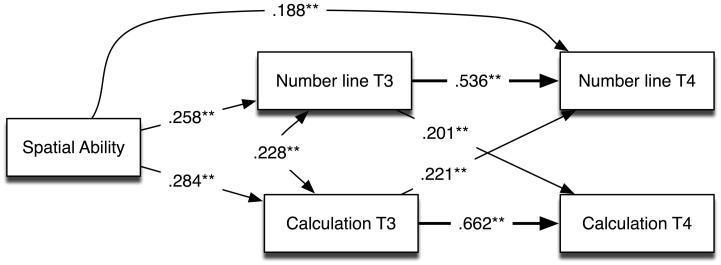
**Path analysis showing longitudinal relations among spatial ability, number line performance, and Calculation (*R*^2^ = 0.356, and 0.584 for number line at T3 and T4, respectively; *R*^2^ = 0.563 and 0.611 for Calculation at T3 and T4, respectively)**. ^**^p < 0.01.

The longitudinal relations among spatial ability, number line performance, and calculation were evaluated using simultaneous path analysis in Mplus (Version 6; Muthén and Muthén, 1998/2011). The β values fit by the model, including all significant direct effects, are shown in Figure 2. The significant indirect effects are listed in Table 3. Significance of the indirect effects was tested using 95% confidence intervals calculated using bias-corrected bootstrap sampling (Geiser, 2013). Although it is not shown in the figure, the regressions controlled for grade and sex for number line and for grade for Calculation.

**Table 3:** **Significant effects (standardized) of spatial ability on number line and calculation at Time 4**.

	**β**	**Confidence intervals**^**a**^	
		**Lower 2.5%**	**Upper 2.5%**
**SPATIAL TO NUMBER LINE TIME 4**			
(a) Total effect	0.389	0.265	0.514
(b) Direct effect	0.188	0.058	0.318
(c) Indirect through number line at T3	0.138	0.047	0.230
(d) Indirect through calculation at T3	0.063	0.011	0.115
**SPATIAL TO CALCULATION TIME 4**			
(a) Total effect	0.240	0.147	0.332
(b) Indirect through number line T3	0.052	0.006	0.097
(c) Indirect through calculation T3	0.188	0.106	0.269

There was no direct effect from spatial ability to calculation at Time 4.

a Confidence intervals were calculated with bias-corrected bootstrapping in Mplus (10,000 samples).

As shown in Figure 2, our data replicated the concurrent and longitudinal relations among spatial ability and number line performance reported by Gunderson et al. (2012), specifically, that growth in number line performance from Time 3 to Time 4 was predicted by spatial ability (i.e., the direct effect from spatial ability to number line at Time 4). There were also significant indirect effects of spatial ability on number line and Calculation performance at Time 4, mediated through Calculation and number line at Time 3 (see Table 3).

These findings extend the results reported by Gunderson et al. (2012) to a slightly older group of children and provide a more complete picture of the longitudinal relations between number line and Calculation because they model the autoregressive effects for both of these variables. Although these results are consistent with Gunderson et al.'s reported pattern of results, the more complete picture shown in the current analysis does not support their larger conjecture that number line knowledge is the critical causal variable that is most relevant for understanding how spatial ability is linked to the development of mathematical skills. The indirect effect of spatial ability on Calculation at Time 4 that is mediated through Calculation at Time 3 (0.188) is significantly larger than the indirect effect mediated through number line performance at Time 3 (0.052, confidence intervals do not overlap). This pattern suggests that it is important to consider a broader model of how spatial abilities may influence the development of mathematical skills.

**Figure 3:**
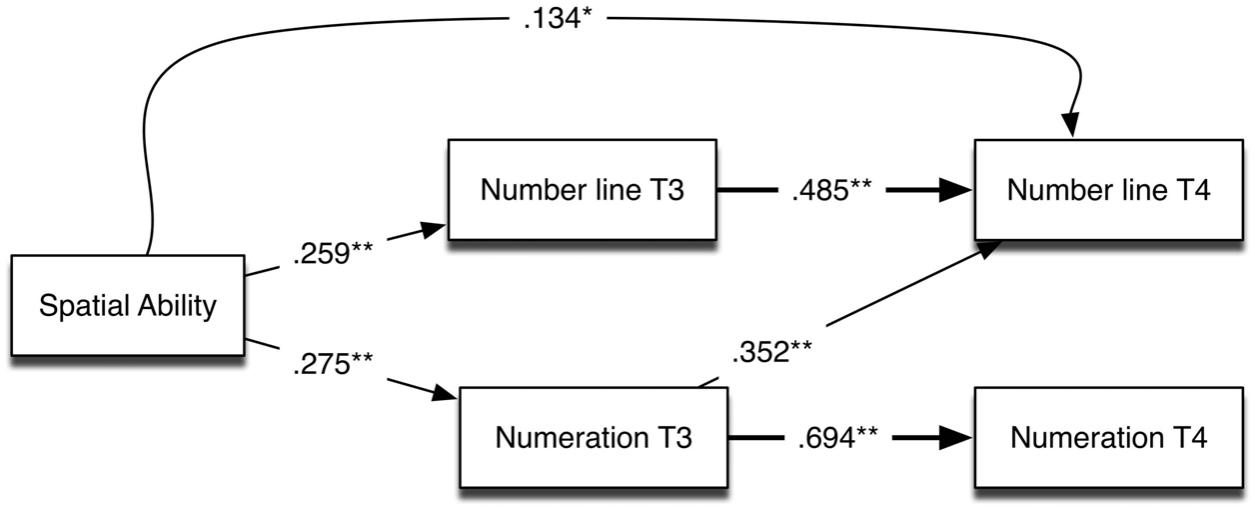
**Path analysis showing longitudinal relations among spatial ability, number line performance, and Numeration (*R*^2^ = 0.351, and 0.617 for number line at T3 and T4, respectively; *R*^2^ = 0.578 and 0.482 for Numeration at T3 and T4, respectively)**. The residual variances between number line and Numeration were not significant at either Time 3 or Time 4. ^*^p < 0.05; ^**^p < 0.01.

We further tested the possibility that the influence of spatial abilities on later mathematical skills is mediated through skills other than number line performance by evaluating and testing a model that included number line and Numeration knowledge (see Figure 3). In this model, control variables included sex and grade for number line at Time 3 and vocabulary and grade for Numeration at Time 3. Note that the Numeration measure, in comparison to the number line task, is a broader assessment of children's understanding of the symbolic number system in the thousands and beyond. As shown in Figure 3, spatial ability was a significant predictor of both number line and Numeration performance at Time 3, and showed a direct link to number line performance at Time 4. In this model, however, it is also clear that the indirect influence of spatial ability on Numeration at Time 4 was mediated only through Numeration at Time 3, because the cross-lagged path through number line at Time 3 was not significant. As shown in Table 4, the indirect effect of spatial ability on growth in Numeration at Time 4 (i.e., mediated by Numeration at Time 3) was significant. Finally, the indirect (mediated) path from spatial ability through Numeration at Time 3 to the number line at Time 4 was significant (see Table 4 for indirect effects), supporting the conclusion that spatial ability is related to the development of mathematical skills via multiple pathways. This model also shows that Numeration, as a measure of number system knowledge, predicts growth in children's number line performance.

**Table 4:** **Significant effects (standardized) of spatial ability on number line and numeration at Time 4**.

	**β**	**Confidence intervals**^**a**^	
		**Lower 2.5%**	**Upper 2.5%**
**SPATIAL TO NUMBER LINE TIME 4**			
(a) Total effect	0.356	0.233	0.479
(b) Direct effect	0.134	0.009	0.258
(c) Indirect through number line T3	0.126	0.041	0.210
(d) Indirect through numeration T3	0.097	0.030	0.164
**SPATIAL TO NUMERATION TIME 4**			
(a) Indirect through numeration T3	0.191	0.088	0.293

There was no direct effect from spatial ability to numeration at Time 4 and no indirect effect through number line at Time 3 and thus, the total effect is indirect through numeration.

a Confidence intervals were calculated with bias-corrected bootstrapping in Mplus (10,000 samples).

## Discussion

How are spatial abilities related to children's mathematical learning? In support of our first hypothesis that spatial abilities are related to children's performance on various mathematical tasks, we found significant correlations between spatial ability and number line task performance, arithmetic, and number system knowledge (Booth and Siegler, 2006; Lachance and Mazzocco, 2006; Gunderson et al., 2012). We also found support for our second hypothesis, that spatial ability predicts growth in number line knowledge. These results were consistent with the results of Gunderson et al. (2012) and extend the link between spatial ability and mathematics to somewhat older children. Performance on the number line presumably requires spatial abilities because, even when children understand the number system range, they still need to determine the approximate location of a number along a continuum and indicate it as an explicit spatial location.

We did not find support for our third hypothesis, that the number line task is specifically predictive of growth in arithmetic knowledge. Gunderson et al. (2012) found that the link between spatial ability (age 5) and symbolic approximate arithmetic (age 8) was mediated by number line task performance. Although the pattern observed by Gunderson et al. was also present in our data, such that the influence of spatial ability on Calculation is mediated through number line performance (i.e., an indirect effect as shown in Table 3), there was also an indirect effect of spatial ability on Calculation at Time 4 that was mediated through Calculation at Time 3 and the latter effect was larger than the former. In other words, number line task performance does not have a privileged role in linking spatial ability to mathematical learning. Instead, we see in Figure 2 that the cross lagged relations between number line and Calculation performance are significant in both directions and of a similar size. These results have multiple interpretations, including (a) the two tasks required overlapping knowledge or skills (e.g., spatial abilities), and/or (b) both are related to performance on some other unmeasured variable or variables. Thus, the present data do not support the strong claim for a central role for number line knowledge in the development of other mathematical skills (see also Sasanguie et al., 2013; cf. Booth and Siegler, 2008; Gunderson et al., 2012).

More generally, in combination with the results of the longitudinal analysis of number line and Numeration knowledge, our results support the view that the number line task is a complex measure that improves with the development of a variety of relevant mathematical and spatial skills. In the cross-lag analysis, number system knowledge was directionally linked to growth in number line task performance. This pattern suggests that understanding of the number system in a range specified in the tested version of the number line task drives improvements in number line task performance (Sasanguie et al., 2013). Ebersbach et al. (2008) also reported that children (5–9 years old) perform linearly on the number line task when the target numbers are those within their counting range. The Numeration measure used in the present research indexes children's grasp of place value structure between 100 and 1000, and thus, reflects the requisite number system knowledge required to perform well on the number line to 1000. The lack of indirect effects from number line performance at Time 3 to Numeration at Time 4 is consistent with the assumption that the Numeration measure indexes number system knowledge that is necessary to perform the number line task, rather than the reverse.

The present findings emphasize that the number line task requires both spatial abilities and number system knowledge (in the range specified by the endpoints of the number line). Children in grades 2 through 5 are still developing their number knowledge in the range to 1000. Many of the children showed a pattern of performance (see Figure 1B), which suggests that their understanding of numbers did not extend past the low hundreds. This pattern is similar to the finding with younger children that their understanding of cardinality does not generalize beyond the number of objects they can count until they gain an understanding of the way in which counting can be used to determine the size of a set (Wynn, 1992; Sarnecka and Carey, 2008). Similarly, children's number naming performance grows gradually as they work with numbers within a certain range (Skwarchuk and Anglin, 2002). In summary, the present data emphasize the important link between number system understanding and the linearity of number line performance.

Given the causal model proposed for the relation between number line performance and Calculation, our finding of joint rather than directional links suggests caution in drawing conclusions about number line task performance as a reflection of children's underlying numerical representations. The overlapping relations between Calculation and number line performance could also reflect the mutual influence that children's conceptual knowledge (i.e., understanding how the number system works in this case indexed by performance on the number line) and specific procedural skills (i.e., the steps that should be taken to solve a mathematical problem) have on each other over the course of the development of their ability to understand and solve arithmetical tasks (Rittle-Johnson et al., 2001; Gilmore, 2006). Booth and Siegler (2008) argued that children's number line task performance reflects their representation of quantity and thus, should be influential in the development of arithmetic skills. They found support for this view when the number line was used in an intervention to represent quantities and to model addition. Kucian et al. (2011) also found transfer from a number line training condition to arithmetic, however, as in the Booth and Siegler intervention, the training condition included both number line and arithmetic practice. Without the explicit training on using the number line as an arithmetic tool, children may not connect number line knowledge to calculation and thus, there may not be a causal link between the two aspects of mathematical knowledge. Instead, the current research suggests that the number line task indexes children's understanding of the number system, and in particular, the ordinal relations among symbolic representations. Other research indicates that number line task performance also reflects children's ability to use proportional reasoning strategies to map these symbolic representations accurately to a specific physical extent, perhaps part of the link with spatial reasoning. Calculation presumably also requires some or all of the same skills, and thus, the two tasks show improvements that are related but are not explicitly directional. Some caution is recommended, therefore, in training number line task performance with the expectation that it will transfer to improved calculation skills. The present results suggest a need for a better understanding of how number line training might provide benefits that are independent of training in specific fundamental skills.

How do the present data advance our understanding of the relations between spatial and mathematical abilities? First, the finding that spatial abilities predict growth in number line task performance replicates and extends the findings of Gunderson et al. (2012) to older children. These results are consistent with the view that spatial abilities are one of the precursor cognitive skills that support children's learning of related mathematical constructs (LeFevre et al., 2010; Mix and Cheng, 2012). The number line task has obvious spatial processing requirements in that the child has to align the numbers according to their place value within two predetermined endpoints of a continuum. Second, our finding that number system knowledge is also a predictor of growth in number line task performance supports a view of the number line task as an index of children's understanding of the number system in a specified range. As shown by Thompson and Opfer (2010), children can show very strong linearity for a number line task in a familiar range, and yet show relatively poor performance on a number line task in an unfamiliar range. To the extent that they use strategies that involve creating an implicit midpoint reference (e.g., at 500 for a 0–1000 number line; Ashcraft and Moore, 2012) and/or apply a proportional reasoning strategy (e.g., 114 is about 10% of 1000, so it is about 1/10th of the distance from the left; Barth and Paladino, 2011), both number system understanding and spatial reasoning are required when children develop strategies and implement resulting procedures to perform the number line task. In summary, it is important to stress that these findings indicate that spatial knowledge is necessary but not sufficient; growth in number line performance is also driven by earlier knowledge of the number system.

To what extent do these data address the issue of whether the number line task reflects children's use of an internal mental representation of number? Our results are neutral on this point as it is not necessary to postulate a specific internal mental representation to understand children's performance on the number line task. It is more parsimonious to assume that the child's task strategy is reflected in the pattern produced (Bouwmeester and Verkoeijen, 2012). Adults show logarithmic patterns in estimation tasks that include large non-symbolic quantities and linear patterns in estimation tasks with symbolic numbers or small non-symbolic quantities (Dehaene et al., 2008). Presumably they are using their conceptual understanding of how the number system is constructed (i.e., understanding of the base-10 structure, that numbers of equal distance are equally spaced, etc.), in combination with proportional reasoning (Barth and Paladino, 2011; Ashcraft and Moore, 2012) to construct a strategy that is suitable for the particular number line with which they are confronted. Children whose initial knowledge about the number system is limited will show increasingly linear patterns of responding as they gain understanding of the number system in the specified range, and as they more skillfully apply their spatial knowledge to construct an appropriate strategy.

Although the cross-lagged correlational analysis has some limitations, it is nevertheless, more stringent that using regression to test for possible directional links over time. One limitation of this method is that performance on both tasks may be related to growth in other skills that were not measured in the current research (i.e., causality may be linked to other variables). Thus, we cannot reject the possibility that future research may show stronger pathways. Nevertheless, the pattern of correlations that was observed between calculation and the number line task did not support the strong hypothesis that number line performance is causally linked to calculation (cf. Gunderson et al., 2012). The view that the number line task is an outcome of children's growing number knowledge, rather than a predictor of it, needs further consideration.

An important methodological issue for future research is the assessment of spatial abilities. The measure of spatial ability in the present study was based on two different tasks (spatial reasoning and spatial memory span) and thus, is presumably better than using a single predictor. However, as noted by Mix and Cheng (2012), further theoretical and empirical work on the construct of “spatial ability” will be necessary to adequately test the various possible links between those abilities and mathematical learning and development. Recent research has identified at least three aspects of spatial abilities that may be important including mental rotation, spatial visualization, and disembedding (i.e., the ability to identify target figures in a distracting background), each of which have found to be correlated with different aspects of mathematical development (Mix and Cheng, 2012). The measures used in the present research reflected these abilities in various degrees (e.g., the spatial reasoning task required some rotation and disembedding and the spatial memory task also required visualization). Other spatial abilities may also be involved in these tasks, and may also be involved in various aspects of mathematics. Future research should address the unique relations of various spatial measures to the development of mathematical skills in young children.

In summary, these findings suggest that growth in performance on the number line task reflects children's knowledge of the number system in the specified range in combination with their ability to apply their spatial abilities to create a successful strategy to solve the task. In contrast to the claims of several other researchers, improvements in number line performance did not appear to be causally linked to improvements in other mathematical skills (cf. Booth and Siegler, 2008; Kucian et al., 2011; Gunderson et al., 2012). Although the present research was not designed to directly investigate the internal representations that might be activated when children perform the number line task, other studies suggest that it is not necessary to assume anything about an internal representation to understand the development of number line task performance (Ebersbach et al., 2008; Barth and Paladino, 2011; Ashcraft and Moore, 2012; Bouwmeester and Verkoeijen, 2012). Thus, it may not be necessary to view performance on the number line task as the reflection of an organized internal knowledge structure that is causally linked to further learning or to categorize number line performance as an index of a fundamental “number sense” (i.e., an internal logarithmic number line). Instead, it may be more useful to view the number line task as a measure of children's ability to skillfully assemble an array of relevant knowledge to perform a complex and (often) novel numerically-relevant task.

### Conflict of interest statement

The authors declare that the research was conducted in the absence of any commercial or financial relationships that could be construed as a potential conflict of interest.
